# Determination of copper and other trace elements in serum samples from patients with biliary tract cancers: prospective noninterventional nonrandomized clinical study protocol

**DOI:** 10.2478/raon-2024-0026

**Published:** 2024-03-30

**Authors:** Martina Rebersek, Nezka Hribernik, Katarina Markovic, Stefan Markovic, Katja Ursic Valentinuzzi, Maja Cemazar, Tea Zuliani, Radmila Milacic, Janez Scancar

**Affiliations:** Department of Medical Oncology, Institute of Oncology Ljubljana, Ljubljana, Slovenia; Medical Faculty, University of Ljubljana, Ljubljana, Slovenia; Jožef Stefan Institute, Ljubljana, Slovenia; Department of Experimental Oncology, Institute of Oncology Ljubljana, Ljubljana, Slovenia; Biotechnical Faculty, University of Ljubljana, Ljubljana, Slovenia; Faculty of Health Sciences, University of Primorska, Izola, Slovenia; Jožef Stefan International Postgraduate School, Ljubljana, Slovenia

**Keywords:** trace elements, copper, predictive biomarkers of response, biliary tract cancer, systemic treatment

## Abstract

**Background:**

Biliary tract cancers (BTCs) are usually diagnosed at an advanced stage, when the disease is incurable. Currently used tumor biomarkers have limited diagnostic value for BTCs, so there is an urgent need for sensitive and specific biomarkers for their earlier diagnosis. Deregulation of the homeostasis of trace elements is involved in the carcinogenesis of different cancers, including BTCs. The objective of the study is to determine/compare the total concentrations of copper (Cu), zinc (Zn) and iron (Fe) and the proportions of free Cu and Cu bound to ceruloplasmin (Cp) and the isotopic ratio of ^65^Cu/^63^Cu in serum samples from healthy volunteers and cancer patients using inductively coupled plasma-mass spectrometry-based methods (ICP-MS).

**Patients and methods:**

In this prospective, noninterventional, nonrandomized study 20 patients and 20 healthy volunteers will be enrolled to identify serum Cu, Zn and Fe levels, Cu isotopic fractionation as a predictive biomarker of response to systemic therapy of BTCs, which will be evaluated by computed tomography. Newly developed analytical methods based on ICP-MS will be applied to metal-based biomarker research in oncology.

**Conclusions:**

In the study the comparison of the total concentration of selected trace elements, the proportion of free Cu and Cu bound to Cp and the isotopic ratio of ^65^Cu/^63^Cu in serum samples from healthy volunteers and cancer patients will be conducted to provide the foundation for the development of a BTC cancer screening methodology and the data on their usability as a potential predictive biomarker for BTCs of response to systemic therapy.

## Introduction

Biliary tract cancers (BTCs) are a heterogeneous group of uncommon and rare epithelial tumors arising from biliary duct cells. Most of them are adenocarcinomas and represent < 1% of all human cancers or 3% of gastrointestinal cancers.^1,2^ Based on anatomical location, BTCs are subdivided into intrahepatic cholangiocarcinoma (ICC), extrahepatic cholangiocarcinoma (ECC) that comprises perihilar cholangiocarcinoma and distal cholangiocarcinoma, and gallbladder carcinoma. This anatomical classification parallels distinct biological and molecular features.^1,2,3,4^

Most patients with BTCs are aged 65 or older.^1,2,3,4^ Mortality rates are approximately 1–2/100,000 for ICC and below 1/100,000 for ECC in most countries.^1^ Increasing mortality from ICC rises was observed globally, due to risk factors and possibly, in part, due to better disease classification.^1^ Mortality from ECC decreases, most likely because of better diagnostics following the increased use of laparoscopic cholecystectomy.^1^ According to the Cancer Registry of Slovenia, there were 223 new microscopically confirmed cases of BTCs in 2020.^5^ Cholangiocarcinoma accounts for 10%−15% of all primary intrahepatic tumors and is the second most common primary liver cancer after hepatocellular carcinoma.^1,3^ The main etiological factors are chronic viral infections (hepatitis B virus and hepatitis C virus), cirrhosis or nonalcoholic fatty liver, obesity, alcohol consumption, tobacco, diabetes mellitus, chronic inflammation of the bile ducts and biliary stasis.^2,3^

Most BTC patients have advanced disease at presentation and relapse despite surgery.^3,4^ Metastatic disease is still incurable, with 5% five-year overall survival (OS) without treatment. The first treatment is surgery of the primary tumor, detected at an early stage in selected patients, and is the only potentially curative treatment, if radically resected (R0) without residual disease.^3,4,6^ Five-year survival ranges depend on the stage of the disease and are from 9% to 25%, 10% to 15% and 15% to 35% for ICC, ECC and gallbladder carcinoma, respectively.^3^ Over the last 10 years, the prognosis of patients has changed significantly, mainly due to the availability of systemic treatment, both adjuvant and, in particular, systemic treatment of metastatic disease with targeted drugs.^3,4,7,8^

The combination of cisplatin and gemcitabine is an approved first-line treatment for unresectable or advanced BTC, with a 30% improvement in overall survival and progression-free survival compared to gemcitabine monotherapy and a statistically significant longer median overall survival for patients on combination therapy.^9^ The phase III TOPAZ-1 clinical trial showed that prior treatment with immunotherapy and chemotherapy in advanced BTCs has a benefit on overall survival.^10^ In the phase III TOPAZ-1 clinical trial, the addition of the anti-PD-L1 immunotherapy durvalumab, to gemcitabine and cisplatin significantly improved survival without additional toxicity compared to cisplatin and gemcitabine combination chemotherapy alone, with a higher objective response rate and longer recurrence-free survival. Thus, combination chemotherapy with cisplatin and gemcitabine in combination with durvalumab immunotherapy is recommended as standard first-line treatment for patients with advanced, metastatic BTC. In recently published results of the phase III KEYNOTE-966 clinical trial in the intention-to-treat population, treatment with anti-PD-1 immunotherapy pembrolizumab in combination with gemcitabine and cisplatin significantly improved the primary endpoint of OS.^4^ At the first interim analysis, treatment with pembrolizumab in combination with gemcitabine and cisplatin did not result in a statistically significant benefit in progression-free survival (PFS). Similar results were obtained in the final analysis for PFS.

Given the current understanding of the biology and the molecular heterogeneity of subgroups of BTCs, it is recommended that extensive molecular genetic profiling be performed prior to the initiation of systemic treatment of advanced metastatic disease.^2,3,4^ Molecular genetic profiling includes microsatellite high instability-high (MSI-H), isocitrate dehydrogenase (IDH1/2) mutations, B-Raf murine sarcoma viral oncogene homolog B (*BRAF*) mutations, human epidermal growth factor receptor 2 (HER2) overexpression or amplification, positive tumors neurotrophic tyrosine kinase receptor (*NTRK*), fibroblast growth factor receptor (*FGRF*) and rearrangement during transfection (*RET*) fusions, as this may allow for personalized, patient-tailored treatment and thus a better prognosis.^2,3,4^

As patients with BTCs are asymptomatic in early stages without both specific clinical presentation and specific serum tumor biomarkers, it is difficult to distinguish BTCs from metastatic disease of other cancers.^2^ Tumor markers can be diagnostic, for tumor screening and early detection, prognostic or predictive for response to treatment. However, widely accepted biomarkers for diagnosing and dynamically monitoring BTCs are still lacking. Currently applied tumor markers carbohydrate antigen 19-9 (CA 19-9) and carcinoembryonic antigen (CEA) have limited diagnostic value because of their low sensitivity and specificity for BTCs.^2,3,4^ CA 19-9 tends to have higher specificity than CEA (92.7% vs. 79.2%, respectively); however, its sensitivity tends to be lower (50% vs. 79.4%, respectively).^2,3,4^ Moreover, they are not specific for gallbladder cancer and can also be significantly elevated in benign diseases of the liver or in other metastatic cancers. When markedly elevated, CA 19-9 is associated with poorer prognosis, and it can also be useful as a predictive biomarker for the tumor’s dynamic changes and thus for response to systemic treatment.^2,3,4^

### Trace elements

Essential trace elements are needed in minute amounts for normal physiology. Among them are iodine (I), copper (Cu), iron (Fe), manganese (Mn), zinc (Zn), selenium (Se), cobalt (Co) and molybdenum (Mo).^11^ Alterations in levels and changes in the expression of proteins involved in metal metabolism have been demonstrated in a variety of cancers. First, the hyperproliferation of cancer cells renders them more reliant on iron than normal cells. Targeting iron metabolism in cancer cells is an emerging field of therapeutics.^12^ When essential trace elements (Mn, Co, Zn, Cu, Se) in the serum, cell fraction, cerebrospinal fluid and tumor tissue samples of malignant brain cancer patients were analyzed, it was shown that elemental profiles in these samples were significantly altered in these cancer patients compared to the healthy individuals. Higher contents of trace elements (particularly Mn, Se, and lead (Pb)) could also be involved in the pathogenesis of brain tumors. Therefore, the urine-to-serum ratio of essential trace elements was proposed as an appropriate diagnostic biomarker in malignant brain tumors.^13^

### Copper

Cu is an essential trace element with a precisely regulated amount in our bodies.^14^ Cu is present in all tissues. It is stored primarily in the liver and then in the muscles, heart, kidneys and brain. In the blood, it is transported bound to the protein Cp.^14,15,16^ It is a coenzyme of many enzymes (e.g., Cu/Zn superoxide dismutase, ceruloplasmin, cytochrome oxidase, tyrosinase, dopamine hydroxylase, lysine oxidase, catalase, selenium-dependent peroxidase, etc.) that are important for cellular respiration and defense against free radicals. It also affects glutathione function. Consequently, Cu deficiency impairs cellular respiration and the regulation of reactive oxygen species. Deregulation of oxidative stress, due to excessive production of reactive oxygen species, impairs cellular DNA repair mechanisms and is an important mechanism in the development of cancer.^14^ In addition to malignant processes, an imbalance of Cu in the body affects the development and progression of chronic, inflammatory and neurodegenerative diseases. Cu deficiency leads to lower overall energy levels, abnormal glucose and cholesterol metabolism, increased oxidative damage, and changes in the function and structure of circulating blood and immune cells.^14^ Cu deficiency is associated with a higher incidence of infections and an increased risk of cardiovascular disease.^14^

Specifically, due to its role in inflammatory and antioxidant processes, Cu has an important role in the development of various cancers, such as gynecological cancers, lung cancer, colorectal cancer and other cancers of the digestive tract.^14^ Recent preclinical and clinical data confirmed that Cu concentrations are abnormal in malignant tissues of mice and in cancer patients. Namely, in humans, elevated Cu concentrations have been found in malignant tissues of the breast, ovary, lung and stomach. Cu is being investigated as a potential target for cancer treatment due to its elevated levels in malignant tissues and its ability to promote angiogenesis, cancer growth and metastasis.^14,15,16,17^ In addition to malignant tissues, the concentration of Cu is also elevated in the serum of cancer patients. Elevated levels of Cu have been measured in the serum of patients with lung cancer, colorectal cancer, epithelial ovarian cancer and biliary tract cancers, and decreased levels in adrenocortical and hepatocellular carcinoma.^14,15,16,17,18,19^

When Cu regulation is disrupted, the quantities and proportions of other essential trace elements may also be altered. Among these, the normal Cu/Zn ratio is known to be disturbed. An imbalance of Cu in the body affects the development and progression of chronic, inflammatory and neurodegenerative diseases and malignant processes. It has been found that high dietary intake of Zn can reduce intestinal absorption of Cu.^20^ Altered intakes of only one of the two (similar observations are also made for the other essential trace elements) may cause an imbalance of the other. For example, relatively low levels of Zn and elevated levels of Cu can increase oxidative stress and impair the antioxidant activity of many enzymes.^20^ Increased Cu/Zn ratios have been found in a wide variety of malignancies, including gastrointestinal cancers, gynecological cancers, breast cancer, and lung cancer, and have been correlated with the stage or condition of the disease at the time of treatment. ^14,15,16,17,18,19,20^ It has been suggested that the Cu/Zn ratio could be used for clinical diagnosis and as a prognostic biomarker to track response to treatment.

### Trace element disorders

Trace element disorders (TEDs) are well established in diseases of genetic origin for which the levels of physiologically relevant metals in the blood are controlled by specific proteins. Inherited TED can result in protein malfunction and therefore, deficiency or toxic accumulation of metal in the body. Well-known examples are Wilson’s disease and hemochromatosis.^21–22^ Diagnosis usually involves gene mutation testing, clinical observations and biochemical testing. Examples of such biochemical tests are determinations of non-Cp-bound Cu, exchangeable Cu, total blood Fe and serum ferritin (light chain). Despite growing evidence that TED are also associated with many types of cancer, knowledge in this field of research is still relatively scarce.^11,12,14,15,16,17,18,19,20^ It requires highly sophisticated interdisciplinary investigations, which promises to provide very useful information on the role of trace metals in cancers.

### Trace element disorders as potential biomarkers in oncology

Several new findings have shown the potential to use TED identification as a biomarker for cancer.^23^ It has been suggested that the imbalance in the Cu/Zn ratio could be used for clinical diagnosis and as a predictive biomarker to track the response to treatment. Cp correlates with immune infiltration and serves as a prognostic biomarker in breast cancer. Elevated serum Cu-Cp levels have been found in lung cancer, colon carcinoma, epithelial ovarian cancer and bile duct cancer, while the expression of Cu-Cp is significantly downregulated in adrenocortical carcinoma and hepatocellular carcinoma.^24^ Serum Cu levels increase in several types of cancer. It was experimentally determined that in hepatocellular carcinoma patients, blood Cu and sulphur (S) are enriched in light isotopes compared with healthy individuals. Isotopic ratios of Cu (^65^Cu/^63^Cu) and S (^34^S/^32^S) were measured to elucidate their use as potential biomarkers of disease.^25^ Changes in the isotopic compositions of Fe, Cu and Zn and their corresponding concentrations in plasma from hematological malignancy patients can be measured to assess their prognostic capability. Imbalances in trace metal concentrations, changes in their speciation and isotopic fractionation need to be further investigated to fully evaluate their emerging biomarker potential.^26^

### Analytical methods

For improvements in cancer therapy efficacy, reliable and optimized analytical and imaging methods using contemporary instrumental techniques that allow investigations on the role of trace elements in cancer, quantitative determination of established or emerging biomarkers (exchangeable and Cp-bound Cu, stable isotope ratio of trace metals, such as Cu, Zn or Fe), as well as the monitoring of penetration, distribution and metabolism of a metallodrug within the target tissue/tumor are needed.^27,28,29,30,31^

Cu toxicity is strongly related to its free (exchangeable) fraction, which is not bound to Cp.^32,33,34^ Due to the important physiological functions and role of Cu in various diseases, it is necessary to quantify its exchangeable and bound to Cp fractions. In clinical practice, Cp in serum or plasma is commonly determined by nephelometry or turbidimetry. They unspecifically measure both holoCp (Cp with Cu) and apoCp (Cp without Cu). The latter Cp form (apoCp) is not relevant for medical diagnosis. To overcome this disadvantage, monolithic chromatography coupled to inductively coupled plasma‒mass spectrometry, which allows simultaneous quantification of exchangeable Cu, Cu bound to human albumin and holoCp, can be used. The chromatographic column used in this method comprises convective interaction media (CIM) affinity and weak anion-exchange disks (Protein G and diethylamine (DEAE) disks) assembled into a single housing forming a CLC monolithic column.^35,36,37,38,39^

Isotopic fractionation of stable isotopes of essential metals was proposed as an emerging predictive biomarker for cancer diagnosis. In hepatocellular carcinoma patients, blood Cu and sulphur (S) are enriched in light isotopes compared with control subjects. Isotopic ratios of Cu (^65^Cu/^63^Cu) and S (^34^S/^32^S) were measured to elucidate their use as potential biomarkers of disease.^31,32^ Changes in the isotopic compositions of Fe, Cu and Zn and their corresponding concentrations in plasma from hematological malignancy patients can be measured to assess their prognostic capability.^31,32^

Trace elements as biomarkers in oncology are promising fields for detecting, diagnosing and predicting responses to treatment. To date, there has been no published clinical trial investigating copper as a predictive biomarker of response to systemic therapy. In this context, we selected patients with locoregional advanced, inoperable or metastatic BTCs for this noninterventional non-randomized prospective clinical trial, treated with first-line systemic chemotherapy or immunochemotherapy, to identify serum Cu levels, its speciation and/or isotopic fractionation as a predictive biomarker of response to systemic therapy in correlation with radiological CT evaluation for response to systemic therapy. The proportion of free Cu and Cu bound to Cp and the isotopic ratio of ^65^Cu/^63^Cu in serum samples will be determined in enrolled healthy volunteers to establish reference values for the general population and to provide the foundation for the development of BTC cancer screening methodology. Determination of the total concentration of trace elements and speciation analysis will be carried out on a quadrupole inductively coupled plasma mass spectrometer (ICP-MS), while isotopic ratios will be precisely determined by multicollector ICP-MS.

## Methods / design

### Study setting

**FIGURE 1.**
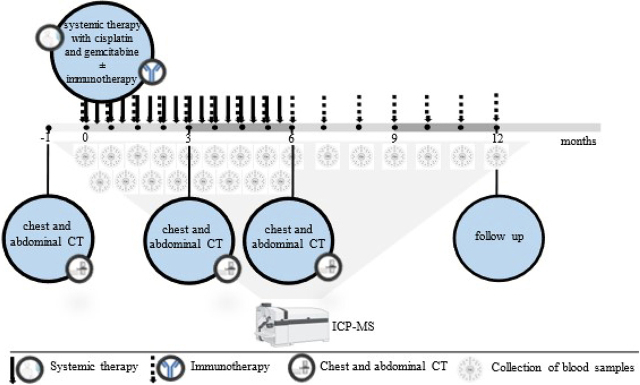
Study flow chart (created with BioRender.com.) ICP-MS = inductively coupled plasma-mass spectrometry-based methods

In a prospective, noninterventional, nonrandomized clinical study started in 2023 at the Institute of Oncology Ljubljana, 20 patients with BTC and 20 healthy volunteers are planning to enroll to provide the development of a BTC cancer screening methodology. The proportion of free Cu and Cu bound to Cp and the isotopic ratio of ^65^Cu/^63^Cu in serum samples will be determined in enrolled healthy volunteers to establish reference values. The inclusion of 20 BTC patients will enable the identification of serum Cu levels as a potential predictive biomarker of response to systemic therapy in correlation with radiological CT evaluation for response to systemic therapy. The clinical study was approved by the Ethics Committee ERIDEK-0095/2022 and the Institutional Review Board ERID-KSOPKR-0091/2022 at the Institute of Oncology Ljubljana, approved 13.12.2022 by the Commission of the Republic of Slovenia for Medical Ethics (0120-472/2022/3). It was registered with ClinicalTrials.gov under the registration number NCT06060990. All patients entering the study signed informed consent forms. Monitoring will be carried out throughout the study. The flow diagram of the study is presented in Figure 1.

### Study outcomes

The main purpose of the research is to determine the total concentration of selected trace elements (Cu, Zn, Fe), the proportion of free Cu and Cu bound to Cp, and the isotopic ratio between ^65^Cu/^63^Cu in blood serum samples of healthy volunteers and BTC cancer patients using methods based on ICP-MS. We will statistically evaluate the results and evaluate the possibilities of using the used analytical methods and the results of these clinical trials in cancer diagnostics and therapy.

Our hypothesis is that serum Cu levels and the ^65^Cu/^63^Cu isotope ratio in cancer patients differ significantly from those in healthy volunteers and that these levels vary according to the response to systemic chemotherapy or chemoimmunotherapy.

### Primary objectives

The first primary objective is to establish reference levels of Cu, Zn and Fe in the serum of healthy volunteers and their levels in locally advanced inoperabile and metastatic BTC patients to establish a framework for reference Cu, Zn and Fe values.

The second primary objective of the study is to identify serum Cu levels, its speciation and/or isotopic fractionation as a predictive biomarker of response to systemic therapy in correlation with radiological CT evaluation for response to systemic therapy.

### Secondary objectives

The secondary objective of the study is to apply newly developed analytical methods based on ICP-MS to metal-based biomarker research in oncology.

### Patient population and recruitment

In this prospective, noninterventional, nonrandomized clinical study, we aim to include 20 patients with locoregionally advanced, inoperable or metastatic BTC who will start with first-line systemic chemotherapy or immunochemotherapy at the Institute of Oncology Ljubljana. Potential study participants will be identified at the institutional multidisciplinary tumor board, comprised of diagnostic radiologists, interventional radiologists, hepatobiliary surgeons, medical oncologists, and radiation oncologists. The study protocol will be explained to all eligible patients in detail. Only those who sign the consent form will enter the clinical study.

Patients’ blood levels of Cu and other trace metals will be determined before starting systemic therapy and at least during one cycle of systemic therapy will be included in the analysis.

Twenty healthy volunteers will also be enrolled, after prior signed written consent to participate in the clinical study, for a single 7 ml blood draw for analysis.

### Eligibility criteria and exclusion criteria

#### Inclusion criteria for patients

- aged ≥ 18 years;
- cytologically or histologically verified BTC;
- no prior systemic therapy and no radiation therapy for advanced, inoperable or metastatic disease;
- WHO performance status 0−2 (ECOG criteria);
- imaging diagnosis (CT of thoracic and abdominal organs) performed within 4 weeks prior to the first administration of systemic therapy;
- disease measurable by RECIST or ECOG criteria;
- signed Consent to Participate in Clinical Research form.

#### Exclusion criteria for patients

- prior systemic treatment and irradiation of inoperable, metastatic disease;
- WHO performance status > 2 (ECOG criteria);
- contraindications for treatment with immunotherapy (known deficiency of the immune system or active immunosuppressive treatment or active autoimmune disease requiring treatment);
- other malignancies, except cured basal cell or squamous cell carcinoma of the skin, carcinoma in situ of the cervix or other cured solid tumors without disease recurrence ≥ 3 years after treatment.

#### Inclusion criteria for healthy volunteers

- aged ≥ 18 years;
- signed Consent to Participate in Clinical Research form.

#### Exclusion criteria for healthy volunteers

- the presence of chronic internal diseases (neurodegenerative, cardiovascular, renal, lung, hematological, gastroenterological diseases) and autoimmune diseases.

### Chemotherapy and immune-chemotherapy regimen

Patients will be followed for up to 12 consecutive months. All patients for whom the medical oncologist decides to be treated with first-line systemic therapy with combination chemotherapy with cisplatin and gemcitabine or combination chemotherapy combined with immunotherapy with the anti-PD-L1 inhibitor durvalumab during the course of treatment will be invited to participate in the clinical trial. Systemic chemotherapy and immunotherapy treatment, duration of treatment and other medical procedures will be performed independently of the study at the discretion of the medical oncologist and according to the recommendations of good clinical practice.

Systemic chemotherapy with cisplatin and gemcitabine will be administered in cycles every 3 weeks, with cisplatin and gemcitabine administered on days 1 and 8 of each cycle, for a total of 8 cycles, or systemic chemotherapy with cisplatin and gemcitabine administered in cycles every 3 weeks, with cisplatin and gemcitabine administered on days 1 and 8 of each cycle, in combination with immunotherapy with durvalumab administered on day 1 of each cycle for a total of 8 cycles, followed by maintenance treatment with durvalumab immunotherapy every 4 weeks until disease progression, unacceptable toxicity or a decision to discontinue treatment by the patient or treating medical oncologist. Standard chest and abdomen CT imaging with contrast will be performed before the start of systemic treatment as baseline imaging to delineate the extent of disease, then 3 months after the start of systemic treatment to assess the efficacy of treatment, and finally 6 months after the start of treatment. All further diagnostic and therapeutic procedures will be part of the standard management of patients undergoing systemic chemotherapy and immunotherapy. All decisions on additional treatment, either surgery or radiotherapy during systemic therapy, will be made by the multidisciplinary gastrointestinal cancer consortium. In the case of adverse events of systemic chemotherapy and immunotherapy, actions will follow standard recommendations for the treatment of complications and discontinuation of systemic treatment. Despite discontinuation of systemic treatment, patients will continue with the planned investigations in accordance with good clinical practice and according to the protocol of the clinical trial, in line with the primary and secondary objectives of the trial.

### Collection of blood samples

All patients will give blood for laboratory tests, blood counts and biochemical tests, including a blood draw to determine the serum Cu level, its speciation and isotopic fractionation before starting treatment and then before each application of systemic therapy. A blood sample will be obtained before the start of systemic therapy and then on days 1 and 8 of each cycle at a regular outpatient check-up at the Institute of Oncology Ljubljana. Patients will additionally have 7 ml of blood drawn into a standard serum tube. The blood sample will be send to the Department of Experimental Oncology of the Institute of Oncology Ljubljana, where the sample will be centrifuged (1300 × g, 10 min, 4°C) and the serum needed for the analysis will be stored at −20°C until the analysis is performed by ICP-MS-based techniques at the Jožef Stefan Institute.

Once patient enrollment in the clinical study has been completed and 20 patients have been enrolled, this will be followed by the enrollment of 20 age- and sex-matched healthy volunteers. The blood sample will be prepared, stored, and analyzed as for the patients.

### Assessment of objective response to the treatment

All patients will undergo diagnostic imaging (chest and abdominal CT with contrast) up to 4 weeks prior to enrollment in the clinical trial and then at 12 (± 7 days) and 24 weeks (± 7 days) after initiation of treatment and if disease progression or adverse events of systemic therapy are suspected. CT scans will be evaluated according to RECIST (response evaluation criteria in solid tumors) and irRECIST criteria (immune-related response evaluation criteria in solid tumors).^40,41^ The IrRECIST criteria divide the response to treatment into different groups: complete response (CR), partial response (PR), stable disease (SD), and progressive disease (PD). Pseudoprogression is defined as transient radiological disease progression in the absence of clinical progression and a progressive reduction in the burden of the underlying disease according to irRECIST criteria in patients who will receive durvalumab immunotherapy in addition to systemic chemotherapy.

### Safety and management of adverse events

In case of adverse events of treatment with systemic chemotherapy and immunotherapy, measures will follow standard recommendations for treatment of complications and interruption of systemic treatment. Adverse events of systemic therapy will be treated in accordance with the recommendations of the NCI Common Terminology Criteria for Adverse Events (CTCAE) v 5.0 and in accordance with good clinical practice.^42^ Despite discontinuation of systemic therapy, patients will continue with planned investigations in accordance with good clinical practice and according to the protocol in the clinical trial according to the primary and secondary objectives of the trial.

Before each cycle of systemic therapy, a laboratory blood sample will be taken as a standard before the decision to continue treatment. Standard peripheral blood sampling will follow hygiene protocols. At the same time, we will add an additional peripheral blood sample to determine Cu in the serum. The collection of additional samples does not pose a major health risk, and the possible complications of blood collection are mainly local: the appearance of a hematoma or infection.^42^ Imaging evaluation poses potential hazards due to contrast agent administration, anaphylactic reaction, and ionizing radiation.^42^

CT imaging diagnostics will take place at the same time intervals as planned for the evaluation of the effectiveness of systemic treatment. In this way, the subjects will not be exposed to additional imaging tests. In the case of a known allergy to the contrast agent, the procedure will be performed with appropriate premedication or with other methods. Hydration and other measures will be taken before the planned imaging diagnostics in case of deterioration of renal function.

### Analytical methodology and blood sample analysis

Total concentrations of trace metals and quantitative determination of relevant species (Cu-Cp, exchangeable Cu) will be determined by ICP-MS or high-performance liquid chromatography coupled to inductively coupled plasmamass spectrometry (HPLC-ICPMS), respectively.^36,37,39^ Emerging metal-based biomarkers (stable isotope fractionation of Cu, and, if relevant, Zn and S) will be followed by multicollector ICP-MS.

### Data analysis

Data from the determination of relevant trace elements (with special attention to Cu) before systemic therapy and at least during one cycle of systemic therapy will be included in the analysis. The association between the change in, for example, Cu concentration, speciation, and/or isotopic fractionation between healthy individuals and those suffering from biliary tract cancer will be statistically analyzed to evaluate the applicability of such an approach as a diagnostic biomarker for disease. The same data will be used to assess response to treatment by a logistic regression model and a multivariate model including different variables. Paired t test or an appropriate nonparametric alternative will be used to compare values at different time points, and analysis of variance (ANOVA) or Kruskal – Wallis’s test will be used when comparing several groups simultaneously. Statistical analysis will be performed using GraphPad Prism (GraphPad, San Diego, CA, USA), and differences will be considered statistically significant if p<0.05.

## Follow-up

All patients are recommended to have a follow-up visit every 3 months in the first 2 years and every 6 months after 2 years after completion of first-line systemic therapy. Follow-up methods will be mainly outpatient visits and hospitalizations. Examinations to be performed on admission will include blood tests for the tumor markers CA19-9 and CEA and CT of the chest, abdomen, and pelvis. Overall survival (OS) will be defined as the time interval from treatment to cancer-related death or final follow-up visit, and OS will be the preferred destination. Progression-free survival (PFS) will be measured from the time of treatment initiation to clinical or radiographic progression or death from any cause.

**FIGURE 2.**
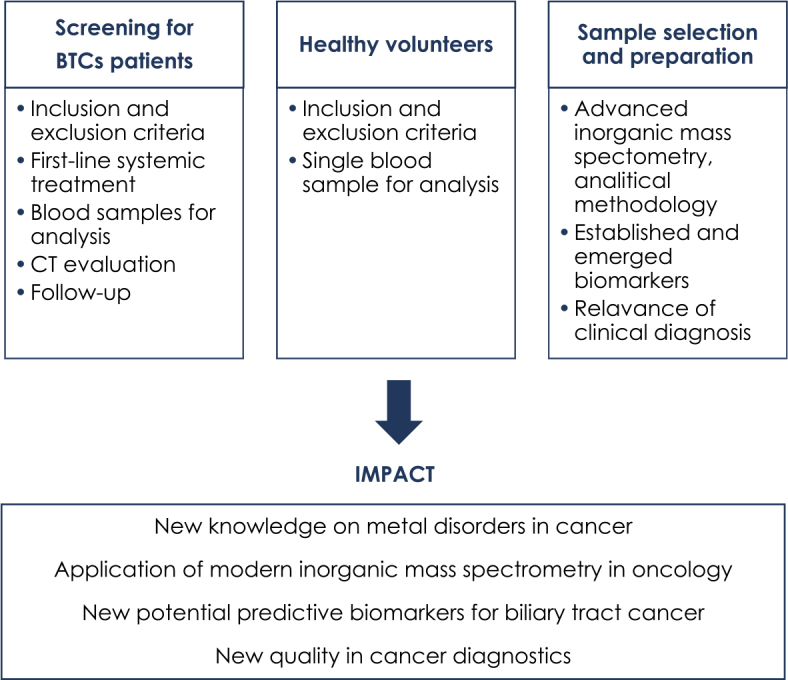
Schematic outline of the proposed clinical protocol.

A schematic outline of the proposed clinical protocol is shown in Figure 2.

### Statistical analysis

Response to treatment will be determined radiologically using RECIST or irRECIST criteria.^40,41^ The objective response to treatment will be calculated as the percentage of patients who have a partial or complete response according to the RECIST or irRECIST criteria among all patients who will receive at least one cycle of systemic therapy and have at least one radiological assessment during systemic treatment. For patients who will not progress or die, the end date for analysis will be the date of last follow-up. Time to disease progression will be defined as the time interval from the date of first therapy administration to the date of disease progression or death, using the Kaplan‒Meier method. Comparison of survival of several groups will be calculated using the log-rank test. The association between the change in Cu concentration, speciation and/or fractionation and response to treatment will be assessed by a logistic regression model and a multivariate model including different variables. Statistical analysis will be performed as previously described in the **Data analysis** chapter.

### Ethics statement

The clinical protocol was approved by the Ethics Committee ERIDEK-0095/2022 and the Clinical Trials Protocol Review Committee ERIDK-SOPKR-0091/2022 at the Institute of Oncology Ljubljana, and 13.12.2022 was approved by the Commission of the Republic of Slovenia for Medical Ethics (0120-472/2022/3). The clinical study will be performed in accordance with the ethical principles of the Declaration of Helsinki. The clinical trial number: NCT06060990. Informed consent will be obtained from each participating patient and healthy volunteer in written form.

## Discussion

BTCs are rare tumors with poor prognosis. Most patients with BTCs have advanced disease at clinical presentation and relapse despite surgery. Metastatic disease is still incurable, with a 5% five-year OS without treatment. In recent years, the prognosis of metastatic patients has changed significantly, with longer median PFS and median OS, mainly due to the availability of systemic treatment, both adjuvant and, in particular, systemic treatment of metastatic disease with targeted drugs.^2,3,4,6,7^ Specific serum biochemical tumor biomarkers are still lacking for the early detection of BTCs, and it is also difficult to distinguish them from metastatic diseases of other cancers.^3,4^ Therefore, extensive efforts are underway to identify more precise biomarkers for the diagnosis, treatment response, and prognosis of BTCs.

Cu, Zn and Fe are among the trace metals that are essential for the normal functioning of the human body.^11,12,13^ They are involved in many biochemical reactions, cofactors of enzymes, and regulate important biological processes by binding to specific receptors and transcription factors. Deregulation of trace metal homeostasis at the cellular and tissue level is a part of the pathology of many cancers. It accelerates the transformation of normal cells into cancerous cells and alters the inflammatory and antitumor responses of immune cells.^11,12,13^

Alterations in the concentrations of Cu and Zn in serum have been widely described in cancer patients.^14,15,16,17,18,19,20^ It has been shown that for several types of cancer, the serum Cu concentration is significantly higher, while that of Zn is significantly lower in patients than in healthy individuals. These differences vary based on various factors (diet, sex, age, type of cancer, etc.) We will also focus on the isotopic fractionation of Cu and, if applicable, Zn in BTC patients to establish a stronger association between the alteration of isotope ratios of these elements and cancer and evaluate the applicability of isotope fractionation as a biomarker of cancer.^27,28,29,30,31^ The hypothesis that the isotopic composition of Cu reflects changes in trace element homeostasis, with higher sensitivity than metal concentrations, will be tested. For this purpose, high-resolution multicollector ICP-MS will be used.^35,36,37,38,39^

Clinical studies with ethical approval will be carried out by a multidisciplinary team from the Institute of Oncology Ljubljana, Slovenia and Jožef Stefan Institute, Ljubljana, Slovenia. Its main objectives are to determine the total concentration of selected essential trace elements (Cu, Zn, Fe), the proportion of free Cu and Cu bound to Cp and the isotopic ratio of ^65^Cu/^63^Cu in blood serum samples from healthy volunteers and locally advanced inoperable and metastatic BTC patients by ICP-MS-based methods.
